# Mutation status concordance between primary lesions and metastatic sites of advanced non-small-cell lung cancer and the impact of mutation testing methodologies: a literature review

**DOI:** 10.1186/s13046-015-0207-9

**Published:** 2015-09-04

**Authors:** James Sherwood, Simon Dearden, Marianne Ratcliffe, Jill Walker

**Affiliations:** Personalised Healthcare and Biomarkers, AstraZeneca, Darwin Building, 310 Cambridge Science Park, Milton Road, Cambridge, CB4 0WGT UK; Personalised Healthcare and Biomarkers, AstraZeneca, Macclesfield, UK

**Keywords:** Non-small-cell lung cancer, Metastatic, Mutation, Biopsy, *EGFR*, *KRAS*

## Abstract

Increased understanding of the genetic aetiology of advanced non-small-cell lung cancer (aNSCLC) has facilitated personalised therapies that target specific molecular aberrations associated with the disease. Biopsy samples for mutation testing may be taken from primary or metastatic sites, depending on which sample is most accessible, and upon differing diagnostic practices between territories. However, the mutation status concordance between primary tumours and corresponding metastases is the subject of debate. This review aims to ascertain whether molecular diagnostic testing of either the primary or metastatic tumours is equally suitable to determine patient eligibility for targeted therapies. A literature search was performed to identify articles reporting studies of mutations in matched primary and metastatic aNSCLC tumour samples. Clinical results of mutation status concordance between matched primary and metastatic tumour samples from patients with aNSCLC were collated. Articles included in this review (*N =*26) all reported mutation status data from matched primary and metastatic tumour samples obtained from adult patients with aNSCLC. Generally, substantial concordance was observed between primary and metastatic tumours in terms of *EGFR, KRAS, BRAF, p16* and *p53* mutations. However, some level of discordance was seen in most studies; mutation testing methodologies appeared to play a key role in this, along with underlying tumour heterogeneity. Substantial concordance in mutation status observed between primary and metastatic tumour sites suggests that diagnostic testing of either tumour type may be suitable to determine a patient’s eligibility for personalised therapies. As with all diagnostic testing, highly sensitive and appropriately validated mutation analysis methodologies are desirable to ensure accuracy. Additional work is also required to define how much discordance is clinically significant given natural tumour heterogeneity. The ability of both primary and metastatic tumour sites to accurately reflect the tumour mutation status will allow more patients to receive therapies personalised to their disease.

## Introduction

Lung cancer is the leading cause of cancer mortality [1], with non-small-cell lung cancer (NSCLC) accounting for ~85 % of primary lung cancers [2]. Metastatic spread of the disease is a complication of advanced NSCLC (aNSCLC) [3], which usually precedes the fatal stages by a few months. Unfortunately, many patients present with metastases at diagnosis [4] due to the relatively asymptomatic earlier stages of the disease.

Increased understanding of the genetic aetiology of aNSCLC over the past decade has provided the opportunity for personalised treatment for some patients, targeting several of the key molecular aberrations now known to be associated with aNSCLC [5, 6]. A recent meta-analysis of the incidence and coincidence of mutations in aNSCLC reported that three genes – tumour protein p53 *(TP53)*, epidermal growth factor receptor (*EGFR)* and v-Ki-ras2 Kirsten rat sarcoma viral oncogenes homolog (*KRAS) –* were commonly mutated in NSCLC of adenocarcinoma (ADC) histology [7], one of the most common histological subtypes of NSCLC [2, 8]. The functional pathways associated with these genes are well-documented [9–11], however in brief: mutations in *EGFR* are known to activate the MAPK/ERK pathway [10, 11]; mutations in *KRAS, BRAF* and *PIK3CA* are known to alter MAPK/ERK activation [10, 11]; and mutations in *TP53* are known to lead to loss of function of this tumor suppressor [9].

Molecular diagnostic testing is now recommended by several clinical guidelines [12–14] for patients with NSCLC to determine eligibility for targeted therapies. For example, *EGFR* tyrosine kinase inhibitors (TKIs), such as gefitinib and erlotinib, are approved for patients with mutations in the *EGFR* gene; it is now widely accepted that response to EGFR TKIs is greater in patients with tumours harbouring *EGFR* mutations compared with wild-type *EGFR* oncogenes [15]. Similarly, translocations involving the anaplastic lymphoma kinase gene predict patients who will respond to the TKI crizotinib [16]. Further to this, new targeted therapies are being developed for patients with other molecular aberrations; for example, selumetinib, cobimetinib and trametinib are being developed for patients with *KRAS* mutation-positive tumours [17].

As a result of the availability of targeted therapies, determining tumour mutation status in patients with aNSCLC is now a key component of diagnosis in many countries, with the hope, where possible, of optimising treatment outcomes [18, 19]. Currently, most aNSCLC cases are diagnosed by a histological analysis of the tumour tissue; for example, around 77 % of patients in England and Wales (UK) are diagnosed in this manner [20]. However, depending on patient ability and/or willingness to undergo sampling, whether samples are available or evaluable, and differing diagnostic practices between countries [12, 13, 21–25], either the primary tumour or a metastatic lesion may be biopsied [26]. However, the concordance in mutation status between matched primary and metastatic tumours is the subject of debate [3, 27–33], with limited understanding as to whether discordance reflects actual heterogeneity in mutations, or is an artifact of technical/sensitivity limitations in testing methodology [34–43]. Nevertheless, given that the collection of multiple invasive samples from a patient with NSCLC is undesirable, it is important to ascertain whether the mutation status of an individual patient’s NSCLC can be accurately characterised from biopsies of either the primary or metastatic sites.

Although research into the concordance of *EGFR* and *KRAS* mutation status between matched primary and metastatic tumours exists [33], to the authors’ knowledge, no review to date has systematically assessed the currently available data regarding whether metastatic samples are representative of primary tumour samples in patients with aNSCLC in terms of multiple mutations, and included consideration of the mutation testing methodologies employed. To address this knowledge gap, we describe in this review the level of mutation status concordance between matched primary and metastatic tumour samples, considering *EGFR, KRAS* and any other molecular aberrations noted in the included literature, as well as describing the mutation testing methodologies used.

## Methods

### Literature search

Literature searches of the MEDLINE® and PubMed® databases were carried out to identify journal articles published before 8 September 2014, which reported studies of mutations in aNSCLC tumour samples of primary or metastatic origin. The following search criteria were used: [NSCLC OR Lung] AND [mutation] AND [Primary] AND [Metas*]. The following were excluded: [non-English papers] AND [Editorials] AND [Commentaries]. Case reports, reviews and meta-analyses were also excluded, due to the variability in mutation testing methodologies used, which would limit the conclusions that could be drawn from such studies.

Articles were reviewed to identify those reporting results of clinical studies of mutation status concordance between matched primary and metastatic tumour samples from patients with aNSCLC. Where possible, the following parameters were recorded: study population demographics (age, gender, ethnicity and smoking status); number of matched tumour samples; description of matched tumour samples; molecular marker assessed; molecular marker assessment technique; mutation frequency in primary compared with metastatic tumour samples; and mutation status concordance rate between primary and metastatic tumour samples. Gene expression data, where reported, were not included.

## Results

### Included studies

**Fig. 1:**
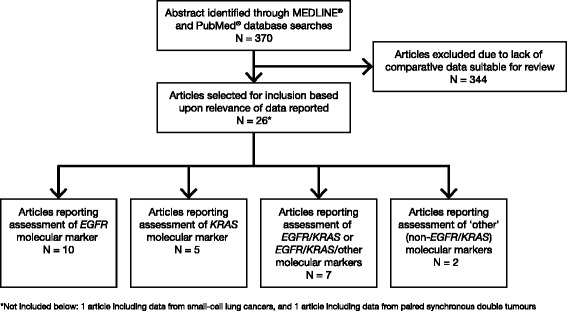
Study selection flow diagram. Literature searches carried out on 25 July 2013, 3 January 2014 and 8 September 2014

The literature search yielded 370 relevant abstracts, of which 26 were considered relevant for inclusion based upon the fact that they reported mutation data (any gene) for both primary and corresponding metastatic samples from patients with aNSCLC (Fig. 1). One study included data from small-cell lung cancers [44], and another included data from paired synchronous double tumours [45]; the remaining 24 studies are listed in Tables 1, 2, 3 and 4.

**Table 1:** Summary of studies reporting assessment of *EGFR* molecular marker

Reference	Patient demographics:	Description of matched pairs				Synchronous/metachronous/metastases, *n*:*n*	Molecular marker assessment technique	Mutation frequency,^c^ *n*/*N* (%)	Concordance, *n/N* (%)
	(i) Median age (range), years	*N*	Tumour sample storage form	Primary	Metastatic: *n*				
	(ii) Gender, *n*/*N* (%) male			*Histological subtype: n*		*Time between primary and metastatic tumour sample collection* ^*b*^			
	(iii) Ethnicity [country^a^]								
	(iv) Smoking status, *n*/*N* (%)								
Assessment of EGFR molecular marker									
Chen *et al.* [27]	(i) 58 (27–84)	180	Archived	Lung	Lymph node: 49	40:140	High-resolution melting method	119/235 (51) vs lymph node metastases 15/49 (31);	[Overall]
	(ii) 112/180 (62.2)				Pulmonary nodules: 41	*N/A*		pulmonary nodules 19/41 (46);	155/180 (86)
	(iii) Asian				Chest wall: 15			distant metastatic tumours 16/35 (46)	[Paired pulmonary primary nodules]
	(iv) Never-smoker: 52/180 (28.9); ever-smoker: 128/180 (71.1)				Pleural: 8				31/41 (76)
					Brain: 5				[Paired primary lung tumours and distant metastases]
					Liver: 3				30/35 (86)
					Adrenal gland: 3				[Paired primary lung tumours and metastatic lymph nodes]
					Retroperitoneal lymph node: 1				44/49 (90)
									[Paired metachronous primary tumours]
									50/55 (91)
Gow *et al.* [28]	(i) 61 (38–80)	67	FFPE	Lung	Brain: 25	N/A	Direct sequencing and ARMS method	[Direct sequencing]	[Direct sequencing]
	(ii) 40/67 (60)				Bone: 20	*Median time between resection of primary and corresponding metastatic tumours: 9.3 months (range: 0–90)*		18/67 (27) vs 26/67 (39)	41/67 (61)
	(iii) [Taiwan]				Pleura/skin/soft tissue: 11				[Direct sequencing and ARMS method combined]
	(iv) Never-smoker: 41/67 (61); ever-smoker: 26/67 (39)				Distant lymph node: 4				49/67 (73)
					Gastrointestinal system: 4				
					Metastatic lung: 2				
					Adrenal gland: 1				
Luo *et al.* [54]	(i) 55 (26–79) at diagnosis	15	FFPE	Lung	Brain	N/A	ARMS method	7/15 (47) vs 8/15 (53)	14/15 (93)
	(ii) 83/136 (61)					*N/A*			
	(iii) [China]								
	(iv) Never-smoker: 73/136 (53.7); ever-smoker: 51/136 (37.5)								
Mansuet-Lupo *et al.* [30]	(i) N/A	10	FFPE	Lung	Lymph node: 8	N/A	‘Locally validated tests’	N/A	10/10 (100)
	(ii) N/A			*ADC: 10*	Pleura: 2	*N/A*			
	(iii) Caucasian [France]								
	(iv) N/A								
Matsumoto *et al.* [53]	(i) N/A (43–70)	8	N/A	Lung	Brain	N/A	Direct sequencing (after laser capture microdissection in some cases)	6/8 (75) vs 6/8 (75)	8/8 (100)
	(ii) 5/8 (63)					*Time between resection of primary and corresponding metastatic tumours (range): 0.5–64 months*			
	(iii) [Japan]								
	(iv) Never-smoker: 3/8 (38); ever-smoker: 5/8 (63)								
Park *et al.* [31]	(i) 61 (32–82)	101	FFPE	Lung	Lymph node	101:0	Direct sequencing and heteroduplex analysis	[Direct sequencing]	[Direct sequencing]
	(ii) 73/101 (72.3)					*[Concurrent]*		21/101 (21) vs 11/101 (11)	89/101 (88)
	(iii) [Korea]							[Heteroduplex analysis]	[Heteroduplex analysis]
	(iv) Never-smoker: 29/101 (28.7); ever-smoker: 66/101 (65.4)							29/101 (29) vs 26/101 (26)	84/101 (83)
Shimizu *et al.* [52]	(i) [Mean] 69.1 (37–83)	70	Paraffin embedded	Lung	Lymph node	N/A	PNA-LNA PCR clamp method	21/70 (30) vs 11/70 (16)	60/70 (86)
	(ii) 46/70 (66)			*ADC: 35*		*[Concurrent]*			
	(iii) [Japan]			*SSC: 24*					
	(iv) Never-smoker: 22/70 (31.4); ever-smoker: 48/70 (68.6)			*ADQCC: 5*					
				*LCC: 4*					
				*Pleomorphic carcinoma: 2*					
Tang *et al.* [60]	(i) N/A	9^d^	FFPE	Lung	Lymph node: 9	N/A	Direct sequencing following laser-capture microdissection	54/56 (96) vs 25/30 (83)	N/A
	(ii) N/A			*ADC: 9*		*N/A*			
	(iii) [USA]								
	(iv) N/A								
Wei *et al.* [50]	(i) [38/50 > 60 years; 12/50 ≤ 60 years]	50	FFPE	Lung	Lymph node	N/A	Real-time fluorescent PCR	50/50 (100) vs 47/50 (94)	(47/50) 94
	(ii) 11/50 (N/A)			*ADC: 49*		*N/A*			
	(iii) Chinese			*SSC: 1*					
	(iv) Never-smoker: 40/50 (N/A); ever-smoker: 10/50 (N/A)								
Yatabe *et al.* [51]	(i) N/A	77	Fresh frozen	Lung	Lymph node	N/A	Direct sequencing	77/77 (100) vs 77/77 (100)	(77/77) 100
	(ii) N/A					*N/A*			
	(iii) [Japan]								
	(iv) N/A								

*ADC* Adenocarcinoma, *ADQCC* Adenosquamous carcinoma, *ARMS* Amplification‐refractory mutation system, *EGFR* Epidermal growth factor receptor, *FFPE* Formalin-fixed paraffin-embedded, *LCC* Large cell carcinoma, *N/A* Not available, *PCR* Polymerase chain reaction, *PNA-LNA* Peptide nucleic acid-locked nucleic acid, *SSC* Squamous cell carcinoma

^a^As described in study (country from which samples were taken from)

^b^Concurrent or non-concurrent if time not specified

^c^Primary vs metastatic tumour samples

^d^56 primary samples and 30 metastatic samples

**Table 2:** Summary of studies reporting assessment of *KRAS* molecular marker

Reference	Patient demographics:	Description of matched pairs				Synchronous/metachronous/metastases, *n*:*n*	Molecular marker assessment technique	Mutation frequency,^c^ *n*/*N* (%)	Concordance, *n/N* (%)
	(i) Median age (range), years	*N*	Tumour sample storage form	Primary	Metastatic: *n*				
	(ii) Gender, *n*/*N* (%) male			*Histological subtype: n*		*Time between primary and metastatic tumour sample collection* ^*b*^			
	(iii) Ethnicity [country^a^]								
	(iv) Smoking status, *n*/*N* (%)								
Assessment of KRAS molecular marker									
Alsdorf *et al.* [55]	(i) N/A	19	FFPE	Lung	Lymph node	N/A	ARMS method with direct sequencing after enrichment of tumour cells by laser capture microdissection	19/19 (100) vs 19/19 (100)	19/19 (100)
	(ii) N/A					*N/A*			
	(iii) [Germany]								
	(iv) N/A								
Badalian *et al.* [57]	(i) N/A (47–76)	11	FFPE	Lung	Bone	N/A	RFLP-PCR	3/11 (27) vs 3/11 (27)	7/11 (64)
	(ii) 8/11 (72.7)					*N/A*			
	(iii) [Hungary]								
	(iv) N/A								
Cortot *et al.* [3]	(i) [Mean] 59.7 (39–73)	21	FFPE	Lung	Brain: 13	6:15	Direct sequencing and mutant-enriched PCR	[Direct sequencing]	[Direct sequencing]
	(ii) 6/21 (28.6)			*ADC: 16*	Lung: 4	*N/A*		3/21 (14) vs 4/21 (19)	15/21 (71)
	(iii) [France]			*SSC: 2*	Bone: 2				[Mutant-enriched PCR]
	(iv) N/A			*LCC: 2*	Soft tissue: 2				17/21 (81)
Holst *et al.* [58]	(i) [Mean] 55.7 (2–72)	10	N/A	Lung	Synchronous/metachronous intrathoracic metastases	Numbers not specified	Topographic genotyping and direct sequencing	6/10 (60) vs 6/10 (60)	10/10 (100)
	(ii) Male:female ratio, 1:1					*N/A*			
	(iii) [USA]								
	(iv) 70 % of patients had a positive smoking history								
Li *et al.* [56]	(i) 60 (36–80)	15	FFPE	Lung	Various	N/A	Oligodeoxy-nucleotide hybridisation of DNA amplified by PCR	5/15 (33) vs 5/15 (33) [present in multiple metastatic sites]	15/15 (100)
	(ii) 13/15 (87)			*ADC: 6*		*[Concurrent]*			
	(iii) [Spain]			*SSC: 4*					
	(iv) Never-smoker: 1/15 (7); ever-smoker: 14/15 (93)			*SCC: 3*					
				*LCC: 2*					

*ADC* Adenocarcinoma, *ARMS* Amplification‐refractory mutation system, *DNA* Deoxyribonucleic acid, *FFPE* Formalin-fixed paraffin-embedded, *KRAS* Kirsten rat sarcoma viral oncogenes homolog, *LCC* Large cell carcinoma, *N/A* Not available, *PCR* Polymerase chain reaction, *RFLP-PCR* Restriction fragment length polymorphism-polymerase chain reaction, *SSC* Squamous cell carcinoma

^a^As described in study (country from which samples were taken from)

^b^Concurrent or non-concurrent if time not specified

^c^Primary vs metastatic tumour samples

**Table 3:** Summary of studies reporting assessment of *EGFR*/*KRAS* or *EGFR/KRAS/*other molecular markers

Reference	Patient demographics:	Description of matched pairs				Synchronous/metachronous/metastases, *n*:*n*	Molecular marker assessment technique	Mutation frequency,^c^ *n*/*N* (%)	Concordance, *n/N* (%)
	(i) Median age (range), years	*N*	Tumour sample storage form	Primary	Metastatic: *n*				
	(ii) Gender, *n*/*N* (%) male			*Histological subtype: n*		*Time between primary and metastatic tumour sample collection* ^*b*^			
	(iii) Ethnicity [country^a^]								
	(iv) Smoking status, *n*/*N* (%)								
Assessment of EGFR/KRAS molecular markers									
Han *et al.* [49]	(i) 60 (44–76)	22	Snap frozen	Lung	Lymph node	N/A	Direct sequencing	[EGFR]	[EGFR]
	(ii) 12/22 (55)			*ADC: 15*		*N/A*		7/22 (32) vs 6/22 (27)	21/22 (95)
	(iii) [China]			*SSC: 7*				[KRAS]	[KRAS]
	(iv) Never-smoker: 6/22 (27); former smoker: 5/22 (23); current smoker: 11/22 (50)							2/22 (9) vs 1/22 (5)	21/22 (95)
Han *et al.* [29]	(i) 66 (40–94)	37	FFPE	Lung	Pleural effusion: 12	32:5	Direct sequencing	[EGFR]	[EGFR]
	(ii) 20/37 (54.1)			*ADC: 37*	Pleura: 9	*N/A*		18/37 (49) vs 16/37 (43)	30/37 (81)
	(iii) [Korea]				Brain: 5			[KRAS]	[KRAS]
	(iv) Never-smoker: 18/37 (48.6); former and current smoker: 16/37 (43.2)				Lymph node: 3			1/37 (3) vs 2/37 (5)	36/37 (97)
					Lung: 2				
					Soft tissue: 2				
					Adrenal gland: 1				
					Pericardial effusion: 1				
					Pericardium: 1				
					Ovary: 1				
Kalikaki *et al.* [46]	(i) 55 (41–70)	25	FFPE	Lung	Lung: 9	0:25	Direct sequencing	[EGFR]	[EGFR]
	(ii) 22/25 (88)			*ADC: 18*	Thoracic wall: 5	*Median time between resection of primary and corresponding metastatic tumours: 30 months (range 4–143)*		5/25 (20) vs 3/25 (12)	18/25 (72)
	(iii) Caucasian			*SSC: 2*	Adrenal gland: 4			[KRAS]	[KRAS]
	(iv) Never-smoker: 3/25 (12); active or former smoker: 22/25 (88)			*ADC/BAC: 2*	Brain: 3			5/25 (20) vs 5/25 (20)	19/25 (76)
				*LCC: 2*	Bone: 2				
				*GCC: 1*	Skin: 1				
					Liver: 1				
Munfus-McCray *et al.* [48]	(i) 56.3 (51–80)	9	FFPE	Lung	Brain: 3	N/A	[EGFR] Bidirectional DNA sequencing	[EGFR]	[EGFR]
	(ii) N/A (66.7)				Lymph node: 3	*N/A*	[KRAS] Pyrosequencing following microdissection of tumour tissue	3/9 (33) vs 2/9 (22)	8/9 (89)
	(iii) [USA]				Pleura: 1			[KRAS]	[KRAS]
	(iv) Never-smoker: 4/9 (N/A); current and former smoker: 4/9 (N/A)				Knee: 1			1/9 (11) vs 2/9 (22)	8/9 (89)
					Contralateral lung: 1				
Sun *et al.* [32]	(i) [Mean] 58 (32–77)	80	FFPE	Lung	Lymph nodes	80:0	Direct sequencing	[EGFR]	[EGFR]
	(ii) 50/80 (62.5)			*ADC: 39*		*[Concurrent]*		21/80 (26) vs 26/80 (33)	73/80 (91)
	(iii) Chinese			*SSC: 31*				[KRAS]	[KRAS]
	(iv) Never-smoker: 31/80 (38.75); ever-smoker: 49/80 (61.25)			*Adenosquamous carcinoma: 6*				1/80 (1) vs 7/80 (9)	74/80 (93)
				*LCC: 4*					
Assessment of EGFR/KRAS/BRAF molecular markers									
Schmid *et al.* [47]	(i) 62 (42–81)	96	FFPE	Lung	Locoregional lymph node	N/A	Direct bidirectional sequencing	[EGFR]	[EGFR]
	(ii) 58/96 (60.4)					*N/A*		4/96 (4) vs 4/96 (4)	90/96 (94)
	(iii) Caucasian [Austria]							[KRAS]	[KRAS]
	(iv) Never-smoker: 22/96 (23); current smoker: 60/96 (63); former smoker: 14/96 (15)							28/96 (29) vs 20/96 (21)	71/96 (74)
								[BRAF]	[BRAF]
								2/96 (2) vs 0/96 (0)	94/96 (98)
Assessment of EGFR/KRAS/p53 molecular markers									
Takahashi *et al.* [61]	(i) [At diagnosis] (43–79)	8	FFPE	Lung	Brain: 7	N/A	High resolution SNP array (following laser capture microdissection in some cases)	[EGFR]	N/A
	(ii) 5			*ADC: 3*	Lymph node: 3	*Time between resection of primary and corresponding metastatic tumours (range): 0–64 months (not reported for 2 sample pairs)*		3/8 (38) vs 3/8 (38)	
	(iii) [Japan]			*SSC: 1*	Liver: 2			[p53]	
	(iv) Never-smoker: 3; ever-smoker: 5			*LCC: 1*	Pulmonary: 1			7/8 (88) vs 7/8 (88)	
				*SCC: 3*	Pleural: 1			[KRAS]	
								0/8 (0) vs 0/8 (0)	

*ADC* Adenocarcinoma, *BAC* Bronchioloalveolar carcinoma, *BRAF* Murine sarcoma viral oncogene homolog B1, *DNA* Deoxyribonucleic acid, *EGFR* Epidermal growth factor receptor, *FFPE* Formalin-fixed paraffin-embedded, *GCC* Giant cell carcinoma, *KRAS* Kirsten rat sarcoma viral oncogenes homolog, *LCC* Large cell carcinoma, *N/A* Not available, *SCC* Small cell carcinoma, *SNP* Single nucleotide polymorphism, *SSC* Squamous cell carcinoma

^a^As described in study (country from which samples were taken from)

^b^Concurrent or non-concurrent if time not specified

^c^Primary vs metastatic tumour samples

**Table 4:** Summary of studies reporting assessment of ‘other’ (non-*EGFR/KRAS*) molecular markers

Reference	Patient demographics:	Description of matched pairs				Synchronous/metachronous/metastases, *n*:*n*	Molecular marker assessment technique	Mutation frequency,^c^ *n*/*N* (%)	Concordance, *n/N* (%)
	(i) Median age (range), years	*N*	Tumour sample storage form	Primary	Metastatic: *n*				
	(ii) Gender, *n*/*N* (%) male			*Histological subtype: n*		*Time between primary and metastatic tumour sample collection* ^*b*^			
	(iii) Ethnicity [country^a^]								
	(iv) Smoking status, *n*/*N* (%)								
Assessment of p16 molecular marker									
Marchetti *et al.* [59]	(i) [Mean] 60 (36–76)	30	FFPE	Lung	Lymph node	N/A	Direct sequencing by PCR-SSCP	6/30 (20) vs 6/30 (20)	(30/30) 100
	(ii) N/A					*N/A*			
	(iii) [Italy]								
	(iv) N/A								
Assessment of somatic alterations									
Vignot *et al.* [62]	(i) N/A (41–82)	15	Frozen	Lung	Locoregional: 7	2:13	Targeted next-generation sequencing assay	[EGFR]	[Somatic mutations]
	(ii) N/A (13/15)			*ADC: 8*	CNS: 3	*N/A*		1/32 (3) vs 1/31 (3)	N/A (94)
	(iii) [France]			*SSC: 3*	Distant adenopathy: 2			[GNAS]	[Passenger mutations]
	(iv) Never-smoker: N/A (1/15); ever-smoker: N/A (14/15)			*LCC: 2*	Adrenal: 1			1/32 (3) vs 1/31 (3)	N/A (63)
				*Basaloid carcinoma: 2*	Cutaneous: 1			[KRAS]	
					Parietal: 1			4/32 (13) vs 4/31 (13)	
								[NOTCH1]	
								1/32 (3) vs 1/31 (3)	
								[PIK3CA]	
								4/32 (13) vs 3/31 (10)	
								[RB1]	
								1/32 (3) vs 1/31 (3)	
								[SMARCA4]	
								1/32 (3) vs 1/31 (3)	
								[STK11]	
								2/32 (38) vs 2/31 (3)	
								[TP53]	
								12/32 (41) vs 12/31 (42)	
								[Large structural alterations]	
								5/32 (16) vs 5/31 (16)	

*ADC* Adenocarcinoma, *CNS* Central nervous system, *EGFR* Epidermal growth factor receptor, *FFPE* Formalin-fixed paraffin-embedded, *KRAS* Kirsten rat sarcoma viral oncogenes homolog, *LCC* Large cell carcinoma, *N/A* Not available, *PCR-SSCP* Polymerase chain reaction-single-strand conformation polymorphism, *SSC* Squamous cell carcinoma

^a^As described in study (country from which samples were taken from)

^b^Concurrent or non-concurrent if time not specified

^c^Primary vs metastatic tumour samples

### Patient demographics

Key demographic data, where available/applicable, are presented in Tables 1, 2, 3 and 4.

### *EGFR* mutation status concordance

In total, 14 reports of *EGFR* mutation status concordance between matched primary and metastatic tumours were identified, of which four were in Caucasian patients: Kalikaki *et al.* [46] (Table 3) analysed 25 primary tumours and corresponding lung (*n* = 9), thoracic wall (*n* = 5), adrenal gland (*n* = 4), brain (*n* = 3), bone (*n* = 2), skin (*n* = 1) and liver (*n* = 1) metastases, and determined the *EGFR* mutation status concordance to be 72 % (18/25). Schmid *et al.* [47] (Table 3) analysed 96 primary tumours and corresponding lymph node metastases of Austrian patients, and found *EGFR* mutation status concordance to be 94 % (90/96). In a US study, Munfus-McCray *et al.* [48] (Table 3) assessed 9 primary tumours and corresponding metastatic tumours of the brain (*n* = 3), lymph node (*n* = 3) or pleura/knee/lung (*n* = 1 each), and found *EGFR* mutation status concordance to be 89 % (8/9). Lastly, Mansuet-Lupo *et al.* [30] (Table 1) analysed 10 primary tumours and corresponding lymph node (*n* = 8) or pleural metastases (*n* = 2) of French patients, and found *EGFR* mutation status concordance to be 90 % (9/10); the discordant result was obtained from a metastatic lymph node containing <15 % tumour cells, and an *EGFR* mutation corresponding to the primary tumour was subsequently identified in another lymph node from this patient, resulting in 100 % concordance.

Other studies of lymph node metastases, a commonly assessed metastatic site, were in Asian patients. Sun *et al.* [32] (Table 3) analysed 80 primary tumours and corresponding lymph node metastases of Chinese patients, and found *EGFR* mutation status concordance to be 91 % (73/80). Two of the discordant cases resulted from a different *EGFR* mutation being present in the primary versus metastatic tumour (E746-A750 vs L747-T751 and L747-P753insS vs R748-P752, respectively). In two more recent studies of Chinese patients, Han *et al.* [49] (Table 3) and Wei *et al.* [50] (Table 1) analysed, respectively, 22 and 50 primary tumours and corresponding lymph node metastases, and found *EGFR* mutation status concordance to be 95 % (21/22) and 94 % (47/50). However, quantitative analysis in Wei et al’s study indicated that *EGFR* mutation ratios (amount of mutant *EGFR*:all *EGFR* present) were significantly lower in metastatic compared with primary tumour samples (Wilcoxon matched-pair test; *P* < 0.01), suggesting a more moderate mutation ratio concordance of 84 %. In two studies of Japanese patients, Yatabe *et al.* [51] (Table 1) and Shimizu *et al.* [52] (Table 1) analysed, respectively, 77 and 70 primary tumours and corresponding lymph node metastases, and found *EGFR* mutation status concordance to be 100 % (77/77) and 86 % (60/70). Park *et al.* [31] (Table 1) analysed 101 primary tumours and corresponding lymph node metastases of Korean patients. *EGFR* mutation status concordance was found to be 88 % (89/101) via direct sequencing, with 11 discordant cases *EGFR* mutation-positive in the primary tumour only and one discordant case *EGFR* mutation-positive in the metastatic tumour only; however, retesting with a more sensitive heteroduplex analysis decreased the concordance to 83 % (84/101).

Another commonly studied metastatic site was the brain. Matsumoto *et al.* [53] (Table 1) analysed 8 primary tumours and corresponding brain metastases of Japanese patients, with an *EGFR* mutation status concordance of 100 % (8/8) detected via direct sequencing. Luo *et al.* [54] (Table 1) implemented the amplification-refractory mutation system (ARMS) method in their retrospective study of *EGFR* mutations in 15 primary tumours and corresponding brain metastases obtained from Chinese patients, which yielded a concordance of 93 % (14/15).

Other studies included additional metastatic sites as well as the brain. Gow *et al.* [28] (Table 1) analysed 67 primary tumours and corresponding metastases of the following sites obtained from Taiwanese patients: brain (*n* = 25); bone (*n* = 20); and pleura/skin/soft tissue, distant lymph node, gastrointestinal system, metastatic lung tumour or adrenal gland (*n* = 22). *EGFR* mutation status concordance was found to be 61 % (41/67) via direct sequencing. The 26 discordant results were *EGFR* mutation-positive in their metastatic tumour only; these were reanalysed using the ARMS method, which indicated that 10/26 (38 %) of these were in fact concordant. Combining the ARMS and direct sequencing results yielded an overall concordance of 73 % (49/67). Han *et al.* [29] (Table 3) analysed 37 primary tumours and corresponding metastases of the following sites obtained from Korean patients: pleural effusion (*n* = 12), pleura (*n* = 9), brain (*n* = 5), lymph node (*n* = 3), lung (*n* = 2), soft tissue (*n* = 2), adrenal gland (*n* = 1), pericardial effusion (*n* = 1), pericardium (*n* = 1) and ovary (*n* = 1); *EGFR* mutation status concordance was found to be 81 % (30/37).

### *KRAS* mutation status concordance

Overall, 10 reports of *KRAS* mutation status concordance between matched primary and metastatic tumours were identified, of which three analysed lymph node metastases. Schmid *et al.* [47] (Table 3, previously described) found *KRAS* mutation status concordance to be 74 % (71/96), including one discordant result of a *KRAS* mutation that was different in the primary (G12C) versus the metastatic (G12R) tumour, and another with unknown mutation status in the corresponding primary tumour of a *KRAS* mutation-positive metastasis. Unusually, two patients with an *EGFR* mutation (one with the mutation in their primary tumour and one with the mutation in a lymph node metastasis) had an additional *KRAS* mutation in the corresponding metastases. Sun *et al.* [32] (Table 3, previously described) found a *KRAS* mutation status concordance of 93 % (74/80). Alsdorf *et al.* [55] (Table 2) analysed 19 primary tumours and corresponding lymph node metastases of German patients. Direct sequencing yielded 4 discordant results; however, re-evaluation of mutation status using a combination of the ARMS method and enrichment of tumour cells by laser capture microdissection found identical *KRAS* mutations in all 19 matched pairs (100 % concordance).

Other studies assessed mixed/non-lymph node metastatic sites, mostly in Caucasian patients apart from Han et al’s study [29] in Korean patients (Table 3, previously described), which found a *KRAS* mutation status concordance of 97 % (36/37).

The remaining studies were as follows: Li *et al.* [56] (Table 2) analysed 15 primary tumours and corresponding metastases of various origin obtained from Spanish patients (with multiple sites tested per patient), and found a *KRAS* mutation status concordance rate of 100 % (15/15). A retrospective study by Badalian *et al.* [57] (Table 2) analysed 11 primary tumours and corresponding bone metastases of Hungarian patients using a restriction fragment length polymorphism-polymerase chain reaction (RFLP-PCR) method, and found a *KRAS* mutation status concordance of 64 % (7/11). Another retrospective study by Kalikaki *et al.* [46] (Table 3, previously described) found *KRAS* mutation status concordance to be 76 % (19/25). Cortot *et al.* [3] (Table 2) analysed 21 primary tumours and corresponding metachronous/synchronous metastases of the brain (*n* = 13), lung (*n* = 4) or bone/soft tissue (*n* = 2 each), obtained from French patients. Direct sequencing found *KRAS* mutation status concordance to be 71 % (15/21); however, re-testing using a mutant-enriched PCR analysis increased the concordance to 81 % (17/21). In a study of US patients, Holst *et al.* [58] (Table 2) analysed 10 primary tumours and corresponding intrathoracic metastases of patients with the bronchioloalveolar adenocarcinoma NSCLC subtype, and found a *KRAS* mutation status concordance of 100 % (10/10). In a further study of US patients, Munfus-McCray *et al.* [48] (Table 3, previously described) found a *KRAS* mutation status concordance of 89 % (8/9).

### Concordance of other mutations

In total, four studies included analysis of other mutations as well as/instead of those in *KRAS* and *EGFR.*

Reichel *et al.* [44] examined the pattern of *p53* mutations in 26 primary lung tumours and 60 corresponding metastases obtained from Swiss patients. A total of 7/9 patients with *p53* mutation in the primary tumour had identical mutations in all corresponding metastases. In one patient with discordant results, a *p53* mutation was found in one metastatic site (liver), but wild-type *p53* was detected in the primary tumour and in a metastatic lesion of the kidney. In the other patient, a *p53* mutation was detected in the primary tumour and one metastatic site (kidney), whereas wild-type *p53* was detected in a metastatic lesion of the liver. Further to this, Holst *et al.* [58] (Table 2, previously described) found that when *p53* loss of heterozygosity was detected in the primary tumour, it was also detected in the corresponding metastases.

An Italian study by Marchetti *et al.* [59] (Table 4) assessed 30 primary tumours and corresponding lymph node metastases, and found a *p16* mutation status concordance of 100 % (30/30).

Schmid *et al.* [47] (Table 3, previously described) observed novel *BRAF* exon 15 mutations in 2 primary tumours and not in corresponding metastases in Austrian patients. However, *KRAS*/*BRAF* and *EGFR*/*BRAF* mutations were found to be mutually exclusive.

### Allelic patterns between primary and metastatic tumours

Two studies included in this review presented results related to *EGFR* mutation heterogeneity in matched primary and metastatic tumour samples.

**Fig. 2:**
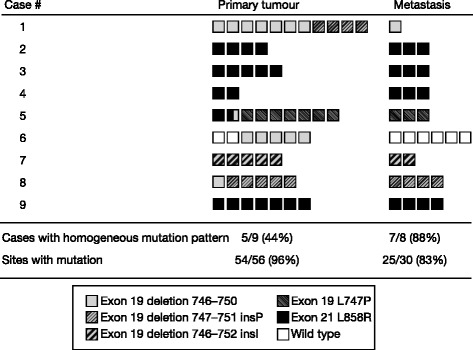
*EGFR* mutation pattern in 56 primary tumour and 30 lymph node metastasis sites obtained from nine patients with *EGFR*-mutant lung adenocarcinomas. A homogeneous mutation pattern was detected in five primary tumours (cases 2, 3, 4, 7 and 9) and all but one (case 6) metastasis case. Case 6 had mixed wild-type and mutant sites in both primary tumour sites and corresponding metastases. EGFR, epidermal growth factor receptor. Reprinted from Cancer Prev Res (Phila), 2008, 1, 192–200, Tang *et al.*, Epidermal growth factor receptor abnormalities in the pathogenesis and progression of lung adenocarcinomas, with permission from AACR [60]

Tang *et al.* [60] (Table 1) assessed *EGFR* mutation heterogeneity in primary tumours and corresponding lymph node metastases from 9 *EGFR* mutation-positive patients, by taking multiple samples from non-contiguous sites of both primary tumours and metastases. Overall, 54/56 (96 %) and 25/30 (83 %) primary and metastatic tumour sites were *EGFR* mutation-positive, respectively. A total of 5/9 patients had identical *EGFR* mutations at multiple sites within the primary tumour and corresponding metastases; however, 2/9 patients presented with two different variants of Exon 19 deletions within the primary tumour: 1/9 patients had a mixture of wild-type *EGFR* and *EGFR* Exon 19 deletions; and 1/9 patients carried both L858R mutations and Exon 19 deletions. However, metastases were non-heterogeneous, with only a single type of mutation detected in each which was always present in at least one site of the primary tumour (Fig. 2).

Further to this, Takahashi *et al.* [61] (Table 3) compared whole-genome allelic imbalance of 8 primary tumours and corresponding brain (*n* = 7), lymph node (*n* = 3), liver (*n* = 2), pulmonary (*n* = 1) and pleural (*n* = 1) metastases obtained from Japanese patients. *p53* and *EGFR* mutations were detected in 7/8 (88 %) and 3/8 (38 %) primary tumours and corresponding metastases, respectively. Genetic alterations were similar between the majority of cases (>67 %); however, there were genetic alterations (specifically, chromosomal regions of allelic imbalance indicated by imbalance of allele homo-/heterozygosity) in the metastatic but not primary tumour in 7/8 cases; and in the primary but not metastatic tumour in 4/8 cases.

### Mutation patterns in multiple primary tumours

Two papers included in our review included analysis of mutation status concordance between multiple primary tumours.

A Japanese study by Matsuzoe *et al.* [45] investigated *p53* mutations in 20 paired synchronous double tumours; *p53* mutations occurred in 7/20 samples, with three different distributions: (i) only one tumour had the mutation (4/7); (ii) each tumour had a different mutation (2/7); and (iii) the same mutation was found in both tumours (1/7). In addition, 3/7 patients had metastatic lymph nodes in which *p53* mutations were found that were identical to those found in the corresponding primary tumour. The third pattern (iii) was suggested to represent metastatic lung cancer.

Chen *et al.* [27] (Table 1) analysed 180 primary tumours and corresponding metastases of Asian patients. They found the following concordance rates for *EGFR* mutations: paired pulmonary primary nodules, 76 % (31/41); paired primary lung tumours and distant metastases, 86 % (30/35); paired primary lung tumours and metastatic lymph nodes, 90 % (44/49); and paired metachronous primary tumours (i.e. diagnosed at different times), 91 % (50/55). Overall concordance was estimated at 86 % (155/180) using a high-resolution melting method.

### Somatic versus passenger mutations

Most studies included in this review were restricted to analysis of a small set of biomarkers. Interestingly, a French study by Vignot *et al.* [62] (Table 4) investigated the presence of multiple somatic alterations in 15 primary tumours and corresponding metastases (locoregional [*n* = 73], central nervous system [*n* = 3], distant adenopathy [*n* = 2], adrenal [*n* = 1], cutaneous [*n* = 1], parietal [*n* = 1]) and determined which alterations were likely to be driving recurrent mutations (defined as mutations that occur in ≥5 % of NSCLC samples in the Catalogue of Somatic Mutations in Cancer or are amplified/deleted in ≥5 % of NSCLC samples in the literature) or passenger mutations (all other mutations). A total of 161 and 190 somatic alterations were identified in the primary and metastatic tumours, respectively. Of these, 159 of these were classed as likely to be passenger mutations; the concordance rate between mutations found in primary compared with metastatic tumours was 94 % for recurrent mutations and 63 % for those considered likely to be passenger mutations.

## Discussion

This literature review aimed to describe the level of mutation status concordance between primary and corresponding metastatic tumours, considering *EGFR, KRAS* and any other molecular aberrations noted. Various factors could contribute to mutation status discordance, such as differences in the sensitivity of mutation testing methods [63], mutation heterogeneity within the samples themselves [60, 61], or evolution of the mutation status of the primary and metastatic tumours [61]. Understanding these factors is important to learn how mutation testing may be improved, in order to ensure that as many patients as possible can access therapies personalised to the mutation status of their NSCLC tumours.

Most studies in this review focussed on *EGFR* and *KRAS* mutations, and in general, substantial mutation status concordance was found in terms of both. Furthermore, there was limited evidence to suggest, as other studies have [64], that *KRAS* mutation status concordance was lower than *EGFR* mutation status concordance in studies that analysed both mutations [29, 32, 46, 48, 49]. *EGFR* mutation frequency was found to be higher than *KRAS* mutation frequency in most [29, 48, 49, 61], but not all [46, 47], studies. A minority of studies included analysis of other molecular aberrations (*BRAF, p53* and *p16*); results of these also indicated substantial mutation status concordance.

Where reported, discordance appeared to be partly related to the mutation testing methodology utilised. Many studies used direct sequencing to assess mutation status, which is known to be relatively insensitive [65], with mutations needing to be present in around 20 % of alleles interrogated [42, 43] to avoid false-negative results [33]. Retesting and confirmation of mutation results with more sensitive techniques was commonly employed by studies in this review. Park *et al.* [31] found *EGFR* mutation concordance was 83 % upon retesting with heteroduplex analysis versus 88 % with direct sequencing; 8 *EGFR* mutations were detected by heteroduplex analysis that were not picked up by direct sequencing, indicating the presence of false negatives. Similarly, the ARMS methodology was employed by some studies, which increased the concordance rates found by direct sequencing [28, 55].

The number of neoplastic versus non-neoplastic cells in tumour samples can also affect detection of mutations [66]. For example, Mansuet-Lupo *et al.* [30] detected an *EGFR* mutation in all metastatic lymph node samples of a patient aside from one sample which contained just 15 % tumour cells. Most studies included in this review used samples with >30 % tumour cells, and some employed laser-capture microdissection [67] to enrich tumour cell content in their samples [53, 55, 61].

Another methodological limitation of most studies in this review was the use of formalin-fixed paraffin-embedded (FFPE) tumour tissues; DNA breakages can occur during formalin fixation [68], with corrupt DNA linked to false-negative or -positive results. Other sample types may be more appropriate for mutation testing, including fresh/archived cytologic samples [69] and blood serum/plasma samples [26, 70] (not included in this review). There are data that have shown *EGFR* concordance rates are higher when analysing archival smear slides compared with FFPE tissues; Sun *et al.* [4] compared primary tumour FFPE histological material with fresh-frozen metastatic material, and found a higher rate of mutation in the fresh samples. In addition to this, a relatively large direct sequencing PCR amplicon was used (292 base pairs), which could have resulted in a much decreased sensitivity in fragmented FFPE-derived material compared with fresh-frozen tissue.

Another potential cause of mutation status discordance was the site of the metastatic sample; only one study included in our review specifically included mutation analysis comparing primary lung tumours and both corresponding lymph node and distant metastases [27], which yielded concordance rates of 90 and 86 %, respectively. This study also compared discordance rates between metachronous and synchronous tumours, which were 16 and 8 %, respectively. There was a lack of similar data from other studies, with most not reporting whether primary and metastatic samples were taken simultaneously.

However, although mutation test methodologies contribute to discordance, natural intratumoural heterogeneity cannot be excluded as a factor, which was observed in some studies in this review. For example, two studies investigated allelic patterns in tumours [60, 61], and found differences in heterozygosity and mutation subtype between primary tumours and their corresponding metastases. When Takahashi *et al.* [61] investigated the nature of each case where different genetic alterations were observed between matched primary and metastatic tumours, the process of metastasis was found to vary, suggesting multiple models of tumour progression and metastatic origins may apply in lung cancer; larger studies are needed to investigate this further. It is also interesting to note the findings of the study by Vignot *et al.* [62], who found that the global level of discordance could at least be partly attributed to passenger mutations.

Although not the focus of this review, limited data were found regarding the association between clinicopathological characteristics and mutation status concordance. Where data were available, as with previous studies, *EGFR* mutations were associated with female sex [32], non-smoker status [32, 47] and ADC histology [32]; one study found *KRAS* was associated with current smokers [47].

*EGFR* mutations in both primary and metastatic tumours were also found to be linked to response to EGFR TKI therapy. For example, Shimizu *et al.* [52] found the disease control rate to be significantly higher in patients with *EGFR* mutation-positive primary and metastatic tumours versus patients with *EGFR* mutation-positive primary tumours only (*P* = 0.062). Furthermore, Kalikaki *et al.* [46] found that of two patients who developed metachronous metastasis following EGFR TKI therapy, one had acquired resistance to the TKI therapy due to a metastatic tumour with a T790M *EGFR* mutation. The T790M *EGFR* mutation is one of the most common mechanisms leading to resistance to TKI therapy, of which there are several [71]. This suggests that consideration of alterations in *EGFR* mutation status during tumour progression is important, and repeat mutation testing may, therefore, be appropriate during clinical management of patients.

Most, but not all, studies included in this review reported that where both primary and corresponding metastatic tumours were *EGFR* mutation-positive prior to any therapy, the same mutation subtype was observed. However, further work is needed to determine the impact of both TKI therapies and chemotherapies on the mutation status of both primary and metastatic tumours (including the emergence of new mutation subtypes), and the resulting need for repeat biopsy.

Furthermore, there is a need for further data reporting on the concordance of the presentation of other biomarkers between primary and metastatic tumours; for example, although mutation expression profiles were beyond the scope of this review, there are ongoing and important developments in personalised therapies targeting proteins differentially expressed in tumours [72].

This review is limited by the heterogeneous patient populations included in the studies, with sample size, patient demographics, disease characteristics, tumour sampling methods and treatment histories differing substantially, and not always reported. This review was also limited to English papers only. Furthermore, until optimum testing techniques have been further researched and defined, the effects of the different mutation testing techniques discussed in this review cannot be fully evaluated.

## Conclusions

The high level of concordance in mutation status between matched primary and metastatic tumours reported in studies here suggest that both sample types are equally viable options for informing treatment decisions based on mutation status. Robust mutation testing must be carried out to ensure accuracy of analysis; key components of robust mutation testing include the sensitivity of the assay and the quality and quantity of the tumour sample used. Furthermore, additional work is required to describe and define how much discordance is clinically relevant given natural tumour heterogeneity. The opportunity, therefore, exists for patients whose primary tumours are not available and/or evaluable to receive personalised therapy following mutation analysis of a metastatic lesion.

## Abbreviations

aNSCLC: Advanced non-small-cell lung cancer
ADC: Adenocarcinoma
ARMS: Amplification‐refractory mutation system
BAC: Bronchioloalveolar carcinoma
BRAF: Murine sarcoma viral oncogene homolog B1
CNS: Central nervous system
DNA: Deoxyribonucleic acid
EGFR: Epidermal growth factor receptor
FFPE: Formalin-fixed paraffin-embedded
GCC: Giant cell carcinoma
KRAS: Kirsten rat sarcoma viral oncogenes homolog
LCC: Large cell carcinoma
N/A: Not available
PCR: Polymerase chain reaction
PCR-SSCP: Polymerase chain reaction-single-strand conformation polymorphism
PNA-LNA: Peptide nucleic acid-locked nucleic acid
RFLP-PCR: Restriction fragment length polymorphism-polymerase chain reaction
SCC: Small cell carcinoma
SNP: Single nucleotide polymorphism
SSC: Squamous cell carcinoma
TKI: Tyrosine kinase inhibitor
